# The role of empathy in antidepressant withdrawal

**DOI:** 10.1177/20451253251397593

**Published:** 2025-11-25

**Authors:** Jim van Schie, Peter Lucassen, Tom Birkenhäger, Sjoerd van Belkum, Mariska Bot, Suzanne Ligthart

**Affiliations:** Department of Psychiatry, VU University Medical Center, Amsterdam, The Netherlands; Department of Primary and Community Care, Research Institute of Health Sciences, Radboudumc, PO Box 9101, Nijmegen 6500HB, The Netherlands; Erasmus MC University Medical Center Rotterdam—Department of Psychiatry, Rotterdam, The Netherlands; Department of Psychiatry, University of Groningen, University Medical Center Groningen, Groningen, The Netherlands; Department of Psychiatry, VU University Medical Center, Amsterdam, The Netherlands; Mental Health, Amsterdam Public Health, Amsterdam, The Netherlands; Department of Primary and Community Care, Research Institute of Health Sciences, Radboudumc, Nijmegen, The Netherlands

**Keywords:** antidepressant discontinuation, depression, empathy

## Abstract

**Background:**

Many antidepressant users experience the process of stopping as challenging because of withdrawal symptoms. Support factors, such as patients experiencing empathy from their healthcare providers, potentially contribute to successful discontinuation.

**Objectives:**

To examine the relation between experienced empathy and successful antidepressant discontinuation.

**Design:**

Part of a larger prospective cohort study in major depressive disorder patients using sertraline or citalopram recruited through university medical centres and connected general practitioners, pharmacies and mental health institutions. The larger study aimed to identify factors associated with remission and patients’ experiences with discontinuing antidepressants.

**Method:**

Patients were followed for 24 months. We measured objective and subjective discontinuation success. Subjective discontinuation was measured with the Discontinuation Success Scale, a scale with three subscales (subjective feeling of success, positive and negative effects of discontinuation). Empathy was measured with the Consultation and Relational Empathy (CARE) measure. To analyse the association between perceived empathy and discontinuation success, we used regression analyses.

**Results:**

Of 918 participants in the larger study, 235 attempted discontinuation and 153 could be included in the analysis. About two-thirds of the participants were successful in discontinuing antidepressant medication. We did not find an association between perceived empathy and objective discontinuation success. Perceived empathy was positively associated with subjective success and negatively associated with the negative effects of discontinuation.

**Conclusion:**

Although we could not demonstrate an association of perceived empathy and objective discontinuation, we consider the finding of a relation between subjective success as relevant because a successful experience regarding antidepressant discontinuation may positively influence the initiation of future attempts.

## Plain language summary

The role of empathy in antidepressant withdrawal

Stopping antidepressant medication is sometimes very difficult because part of the patients experience serious adverse effects when stopping, leading to unsuccessful withdrawal. Apart from the technical aspects of withdrawal (dosage, speed) non-specific factors are important for successful discontinuation. Non-specific factors are for example the quality of the relation between patient and professional or the patients’ expectations prior to the discontinuation process. An important element in the quality of the relation is the empathy patients experience from the healthcare professional. We were able to study the role of empathy in a subsample of a larger study; 153 patients attempting to discontinue their antidepressant. Of the 153 participants 98 were successful in stopping the antidepressant medication. We found no relation between perceived empathy and successful stopping the antidepressant medication. We did find a relation between perceived empathy and negative symptoms experienced during the discontinuation process: the higher the experienced empathy, the less bothersome the discontinuation process. We recommend an empathic attitude from healthcare professionals towards their patients when they attempt to stop the antidepressant. We also recommend further research into other elements of the quality of the relation (genuineness, trust, feeling of alliance) and expectations of patients prior to stopping).

## Introduction

Antidepressant medication is widely used for the treatment of major depressive disorder (MDD) and anxiety disorders.^1^ In line with the global trend, antidepressant prescriptions have been rising steadily in the Netherlands (1 million users in 2021),^2,3^ as is long-term use: 30.3% over the period 1995–2005 and 43.7% in 2005–2015,^4^ stabilising since then with 150.000–200.000 users.^5^ The use of antidepressants is associated with high societal expenses.^6^ Studies have shown that 30%–50% of the long-term antidepressant users have no evidence-based indication to continue this medication.^7–10^ Many antidepressant users experience the process of stopping as challenging because of withdrawal symptoms, also known as antidepressant discontinuation syndrome.^11,12^ The incidence of at least one withdrawal symptom is reported to be 31% after antidepressant withdrawal versus 17% after discontinuation of placebo treatment^13^; for the incidence of severe withdrawal symptoms, the percentages are 2.8% and 0.6%, respectively.^13^ However, these data are probably an underestimation of the frequency and severity of withdrawal effects as the data were from efficacy trials in which withdrawal symptoms were a minor issue and registration of these symptoms was often based on spontaneous reports.^14^ Other recent evidence, including observational studies with selected populations, reported incidence rates for withdrawal effects between 50% and 60%.^15^

Biological factors play an important role in the development of withdrawal symptoms. Arguments are, for example, that discontinuing antidepressants with short half-lives causes withdrawal symptoms more often than antidepressants with long half-lives and that selective serotonin reuptake inhibitors (SSRI) serum levels before and just after withdrawal predict increased withdrawal symptoms.^16^ The role of psychological factors is less clear. Previous research on antidepressant discontinuation success has predominantly focused on the sociodemographic and clinical characteristics of patients, but no consistent pattern of predictors has been found.^17^ Patients thinking about withdrawal fear relapse or withdrawal symptoms; negative experiences in the past increase these fears, hindering discontinuation.^18–21^ Patients have also experienced a lack of knowledge and support in professionals when they attempt to discontinue antidepressants.^22,23^ As a nocebo effect, these experiences contribute to the burden of withdrawal symptoms. Patient experiences and professional support factors, such as therapeutic alliance and empathy, have received less attention, while these could be relevant for successful discontinuation.^22–26^ Importantly, successful discontinuation of antidepressants and antipsychotics was predicted by good therapeutic alliance with the prescribing physician (characterized by agreement on treatment goals and mutual engagement).^27^ This study did, however, not examine the effect of the related construct empathy of the healthcare provider^28^ (understanding the patient, expressing this understanding and acting according to the understanding), while in general empathy enhances care experiences and outcomes, including positive effects on anxiety and depression.^29–31^

We assume that healthcare providers’ empathy could play an important role in the process of discontinuation of antidepressants. Therefore, this study aims to examine the relation between empathy that patients experience from their healthcare providers and the success of an antidepressant discontinuation attempt.

## Methods

### Design

This study is part of the multicentre OPERA-monitor study (Netherlands study of Optimal PERsonalized Antidepressant use), a prospective cohort study, exploring the duration of antidepressant use until remission in MDD patients, identifying factors associated with remission and investigating patients’ experiences with discontinuing antidepressants in patients using citalopram or sertraline. Participants in this study were recruited between June 2019 and December 2022. OPERA-monitor initially functioned also as an entry cohort for an antidepressant discontinuation trial, but this trial was stopped preliminary due to insufficient patient enrolment. The reporting of this study conforms to the Strengthening the reporting of observational studies in epidemiology (STROBE) statement.^32^

### Participants

Participants were recruited through university medical centres and connected general practitioners (GPs), pharmacies and mental health institutions. They completed online questionnaires every 2 months and were followed for 24 months. In addition to the recurring questionnaires, participants received a one-time, questionnaire regarding attempts to discontinue antidepressants. Inclusion criteria were patients aged 18–75 years, using citalopram or sertraline <18 months for depression, and who did not yet reach stable remission. The use of citalopram or sertraline was determined by the fact that OPERA-monitor was planned as an entry cohort for a discontinuation trial. Exclusion criteria were: bipolar disorder, psychotic disorder, the use of antidepressants other than sertraline and citalopram, except the use of a low dose of mirtazapine (max. 15 mg) or amitriptyline (max. 25 mg), addiction to alcohol or other substances, insufficient command of Dutch language, ever being admitted to a psychiatric hospital for depression and having had more than three prior treatments for depression, consisting of at least two antidepressant treatments lasting for 6 weeks each. The exclusion of patients using other antidepressants than citalopram or sertraline was determined by the OPERA-discontinuation trial. In total, 918 participants were included. For this article, we used a subsample of the participants in OPERA-monitor fulfilling the following criteria: responding to the additional questionnaire about attempts to discontinue antidepressants, ever attempting to discontinue antidepressant use, completing the Discontinuation Success Scale (DSS) and the Consultation and Relational Empathy (CARE) measure (*n* = 153).

### Measures

Sociodemographic variables, including age, sex, marital status, education and nationality, were collected in the baseline questionnaire. In this questionnaire, we also assessed the following clinical characteristics: baseline depression score (inventory of depressive symptomatology (IDS)), number of lifetime depressive episodes, cumulative duration of depression, type and dosage of the current antidepressant, self-reported psychiatric comorbidity and other medications. Dose was expressed in under, at or above minimum effective dose (MED): 20 mg/day for citalopram, 50 mg/day for sertraline.

#### Perceived healthcare provider empathy

Healthcare provider empathy, as perceived by participants, was measured using the validated Dutch version of the CARE measure in the additional questionnaire.^33,34^ In this measure, patients are asked to rate their perception of the empathy of the healthcare provider with whom they most recently discussed antidepressant discontinuation. The CARE measure consists of 10 questions using a five-point Likert scale ranging from “poor” to “excellent.” The overall score of the CARE measure is the sum of all ten items (10–50). Participants with ⩾3 missing responses in the CARE measure were excluded from the analyses (*n* = 3).^33^ When participants had fewer than three missing responses, we imputed the mean of the other item scores (*n* = 15).

#### Discontinuation success

Discontinuation success was measured in the one-time questionnaire. We distinguished objective and subjective discontinuation success.^35^ We assessed objective discontinuation by asking patients, “Have you succeeded in quitting taking antidepressants?,” resulting in a dichotomous outcome (yes/no). For the assessment of subjective discontinuation success, we used the DSS, which was translated from the English version into Dutch and checked with backward translation. The DSS is a 24-item self-report questionnaire with three subscales: subjective feeling of success (six items), positive (nine items) and negative (nine items) effects of discontinuation.^35^ All items are rated on a five-point Likert scale ranging from “do not agree at all” to “agree completely.” Items 4 and 5 (part of the success subscale) were reversed prior to calculation of subscale sum scores. Higher sum scores on the success, positive and negative effects subscales indicated more subjective success, more positive effects and more negative effects of discontinuation, respectively. The DSS is reliable, has good construct validity and criterion validity with objective discontinuation and subjective well-being.^35^

### Statistical analysis

We used descriptive statistics to summarize the characteristics of the participants and to determine the level of perceived empathy and the outcomes of objective and subjective discontinuation success. We employed Pearson point-biserial correlation to examine the correlation between objective success and the DSS subscales. We considered values between 0.4 and 0.7 as moderate.^36^ We calculated Cronbach’s alpha as a measure of internal consistency (reliability) in the sample included in the analysis.

To analyse the association between the independent variable ‘perceived empathy’ and the dependent variable ‘discontinuation success’, logistic regression analyses were conducted for the outcome ‘objective discontinuation success’ and linear regression analyses for each subjective discontinuation subscale. Based on previous research, the influence of several sociodemographic and clinical variables was assessed. Model 1 was unadjusted, model 2 adjusted for sociodemographic variables and model 3 additionally adjusted for clinical variables. To prevent issues with multicollinearity and to ensure parsimony, only the most relevant variables were included as covariates in the regression models.^37^ Preliminary analyses were performed to assess assumptions, including linearity, normality, multicollinearity, homoscedasticity and absence of outliers. The discontinuation attempt may have occurred before participation in this study, which could introduce recall bias. Therefore, all regression analyses were repeated in a subgroup that had attempted discontinuation in the previous 2 years. This was based on the following question in the one-time questionnaire: “did you try to discontinue your antidepressant in the past 2 years (yes/no)?.”

*p*-Values of less than 0.05 were considered statistically significant. All analyses were conducted using IBM SPSS Statistics (version 28) (IBM Corp., Armonk, NY, USA).

## Results

**Figure 1.**
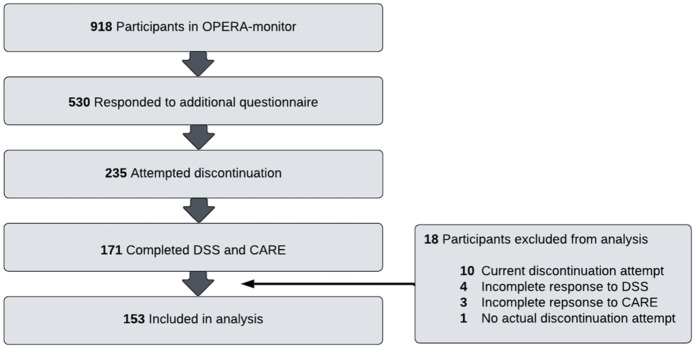
Study flow diagram.

Figure 1 shows the flow chart of the participants. About two-thirds of the 153 included patients were female; the mean age of the participants was 44.4 years (Table 1). Most participants (*n* = 101) used citalopram, the other 52 used sertraline (Table 1). About one fifth of the participants used a dosage below the minimum effective dose. Of the participants, 61% had psychiatric comorbidity; 44% of the participants used other medications. Self-reported lifetime comorbidity consisted mainly of anxiety disorders (*n* = 60), posttraumatic stress disorder (*n* = 32), obsessive compulsive disorder (*n* = 12, eating disorder (*n* = 11) and other psychiatric diagnoses (*n* = 20). Participants included in this study (*n* = 153) did not differ from the participants not included (*n* = 765) concerning sex, education, marital status, type of antidepressant, antidepressant dose, lifetime duration of depression, psychiatric comorbidity and comedication; the included participants were older with a mean age of 44.4 versus 40.7 (Supplemental Table 1).

**Table 1.** Sociodemographic and clinical characteristics of the sample of participants (*n* = 153).

Sociodemographic and clinical characteristics	Mean (SD) or *N* (%)
Age (years)	44.4 (14.8)
Sex (female)	106 (69.3%)
Nationality	
Dutch	148 (96.7%)
Other	5 (3.3%)
Marital status	
Married, living together or in a relationship	103 (67.3%)
Single, divorced or widow	50 (32.7%)
Education level	
Lower	18 (11.8%)
Medium	61 (39.9%)
High	74 (48.4%)
Depressive episodes during lifetime	
1	44 (28.8%)
2	49 (32.0%)
⩾3	60 (39.2%)
Cumulative duration of depression (months)	
<12	41 (26.8%)
12–36	68 (44.4%)
>36	44 (28.8%)
Type of antidepressant	
Sertraline	52 (34.0%)
Citalopram	101 (66.0%)
Prescribed dosages (MED)	
Below MED	33 (21.6%)
At MED	80 (52.3%)
Above MED	40 (26.1%)
Psychiatric comorbidities	
0	59 (38.6%)
1 to 2	63 (41.2%)
⩾3	31 (20.3%)
Comedications	
0	85 (55.6%)
1 to 2	41 (26.8%)
⩾3	27 (17.6%)
Number of discontinuation attempts	
1	118 (77.1%)
2 or more	35 (22.9%)

Lower: No, primary, pre-vocational, junior or preparatory or short secondary vocational, lower general secondary education; Medium: senior general secondary education, pre-university education, secondary vocational education; High: higher professional education, university, PhD.

MED, minimum effective dose; SD, standard deviation.

### Perceived empathy

Participant responses to the CARE measure had a mean score of 37.6, a score between “good” and “very good” (Table 2). The lowest score was 13, and the highest 50. The GP was most frequently mentioned as the last healthcare provider that patients spoke to about discontinuing antidepressant use (48.4%).

**Table 2.** Descriptive statistics of the measured variables of the sample of participants (*n* = 153).

Variables	Min	Max	Mean (SD)
CARE measure	13	50	37.6 (8.9)
DSS success	6	30	21.8 (6.5)
DSS positive effects	9	45	29.1 (8.2)
DSS negative effects	9	44	23.5 (8.7)
Objective success		Successful *N* (%)	Unsuccessful *N* (%)
		98 (64.1%)	55 (35.9%)

CARE, Consultation and Rational Empathy Measure; DSS, Discontinuation Success Scale.

### Antidepressant discontinuation

For objective discontinuation, 98 participants were successful, and 55 were unsuccessful (Table 2). Among the unsuccessful participants, 24 still took their antidepressant in the same dose, 18 switched to another antidepressant, 9 took their antidepressant in a lower dose and 4 took their antidepressant in a higher dose. Concerning subjective discontinuation, mean sum scores on the subscales were 21.8 for the DSS success, 29.1 for the DSS positive effects and 23.5 for the DSS negative effects (Table 2). Cronbach’s alphas were 0.82 (DSS success), 0.89 (DSS positive effects) and 0.90 (DSS negative effects).

There was a moderate relation between subjective and objective success outcomes. Both the DSS success subscale and the DSS positive effects subscale demonstrated significant positive correlations with objective discontinuation success (Pearson *r*_pb_ = 0.609, *p* < 0.001; Pearson *r*_pb_ = 0.406, *p* < 0.001, respectively). The DSS negative effects showed a significant negative correlation with objective discontinuation success (Pearson *r*_pb_ = −0.447, *p* < 0.001 respectively).

### Perceived empathy and discontinuation success

A significant positive association was found between perceived empathy and DSS subjective success scores in a univariate regression model, with CARE as the independent variable and the DSS success score as the outcome variable (*b* = 0.138, 95% CI (0.023–0.254), *p* < 0.05). When sociodemographic and clinical characteristics were included in the regression model, the association remained significant (*b* = 0.129, 95% CI (0.009–0.249); see Table 3.)

**Table 3.** Overview of linear and logistic regression models with the CARE score as an independent variable and DSS success, DSS positive effects, DSS negative effects and objective success as outcome variables.

Regression models	DSS success			DSS positive effects			DSS negative effects			Objective success		
	*B*	CI (95%)	*p*	*B*	CI (95%)	*p*	*B*	CI (95%)	*p*	OR	CI (95%)	*p*
Model (covariates)												
CARE score	**0.138**	**0.023 to 0.254**	**0.019**	**0.155**	**0.009 to 0.301**	**0.037**	**−0.203**	**−0.356 to −0.050**	**0.010**	0.993	0.956 to 1.030	0.698
CARE score (+ demographic)	**0.133**	**0.015 to 0.251**	**0.028**	0.140	−0.007 to 0.288	0.061	**−0.188**	**−0.342 to −0.035**	**0.017**	0.989	0.951 to 1.028	0.560
CARE score (+ demographic + clinical)	**0.129**	**0.009 to 0.249**	**0.035**	0.144	−0.007 to 0.295	0.062	**−0.173**	**−0.326 to −0.021**	**0.026**	0.986	0.946 to 1.027	0.493

Demographic covariates: sex, age, marital status and education level; clinical covariates: type of antidepressants (AD), dosage compared to MED, cumulative duration of depression, number of psychiatric comorbidities and number of comedications. Significant effects are printed in bold.

*B*, regression coefficient; CARE, Consultation and Rational Empathy Measure; CI, confidence interval; MED, minimum effective dose; OR, odds ratio; *p*, *p*-value.

The relation between perceived empathy and DSS positive effects scores was also significant in the univariate model (*b* = 0.155, 95% CI (0.009–0.301)), but this association disappeared when sociodemographic and clinical variables were added to the model (*b* = 0.144, 95% CI (−0.007 to 0.295)). Despite the loss of statistical significance, the regression coefficient and confidence interval remained relatively consistent across the models.

For the DSS negative effects, we identified a significant negative association with perceived empathy. This indicates that an increase in perceived empathy is associated with a decrease in negative symptoms of discontinuation (unadjusted *b* = −0.203, 95% CI (−0.356 to −0.050); adjusted *b* = −0.173, 95% CI (−0.326 to −0.021)).

Logistic regression analyses showed no association between perceived empathy and objective discontinuation success. Both the univariate model and the multivariate models did not reveal a statistically significant association (OR = 0.993, 95% CI (0.956–1.030) and OR = 0.986, 95% CI (0.946–1.027), respectively).

### Subgroup analysis

Of 153 patients, 107 tried to quit their antidepressant medication in the past 2 years. Supplemental Table 2 shows the results of the regression analyses in this subgroup. In both unadjusted and adjusted models, we again found significant positive associations of perceived empathy with DSS success and significant negative associations between perceived empathy and DSS negative effects, whereas we now also observed a significant positive association between perceived empathy and DSS positive effects. Similar to the total group analyses, no association was found between perceived empathy and objective discontinuation success.

## Discussion

### Summary

This study showed that about 2/3 of the participants treated with sertraline or citalopram for depression were successful in discontinuing antidepressant medication. No significant association was observed between perceived empathy and objective discontinuation success. Perceived healthcare provider empathy had a small, significant positive association with subjective discontinuation success. Higher scores on the CARE measure (i.e., higher perceived healthcare provider empathy) were associated with greater subjective success and fewer negative effects of antidepressant discontinuation.

### Limitations

This study has several strengths. First, we utilized validated tools, ensuring valid and reliable data. Second, recruitment of participants through various settings, including GPs, mental healthcare institutes and pharmacies, reduced selection bias and enhanced the heterogeneity of our sample, ensuring relevance and applicability to everyday clinical practice.

A limitation of this study was, first, the observational design, which does not enable us to establish causality. Second, a possible explanation for the lack of association between perceived empathy and objective discontinuation success is the insufficient statistical power of the logistic regression analysis. In addition, a significant part of antidepressant users discontinues their medication without caregiver support, which may have diluted the effect of caregiver empathy.^38^ We also did not have data about how patients discontinued and whether they decided to discontinue themselves. Third, although we translated the English version of the DSS into Dutch and then back into English, we did not assess the content validity of the Dutch DSS using cognitive interviewing; we have investigated the reliability and validity of the Dutch DSS to a limited extent. Fourth, more clarity would have been established if we had had more information about the circumstances, underlying reasons and expectations of participants who lowered the dosage or switched to another antidepressant. Fifth, the OPERA study focused on two of the most widely used antidepressants with the lowest rates of withdrawal symptoms.^13^ Therefore, the results of this study cannot be generalized to other SSRIs. Sixth, we had insufficient data to address the fact that relapse of depression could be a manifestation of the antidepressant discontinuation syndrome.^39^ Seventh, as our study was a secondary analysis of the larger OPERA-monitor study, we did not calculate a sample size. Finally, for the same reason, we did not measure any non-specific factors other than empathy. This is certainly relevant for expectations. Negative expectations concerning the outcome of a withdrawal attempt, based on prior experience or induced by communication from health professionals, could negatively influence the outcome of the attempt.^40^

### Comparison with existing literature

As stated earlier, there are arguments for the role of physiological mechanisms in the development of withdrawal symptoms, indicating the importance of the discontinuation method.^16,41–43^ However, there is ample evidence that any physical intervention depends not only on its mechanism but also on the context of delivering the intervention. Medication side effects are caused not only by their pharmacological properties but also by expectation: patients expecting side effects will experience more side effects than patients not expecting side effects.^44^ This process will also play a role in the experience of withdrawal symptoms. These expectations can be initiated by the informed consent procedure of antidepressant withdrawal or by patients who are familiar with other patients who experienced withdrawal effects. Expectation is a mechanism that is strongly influenced by the context of delivering the treatment. Factors influencing patient expectation are for example the quality of the relation with the patient (therapeutic alliance, genuineness and empathy).^24,25^ Patients consider the quality of the relationship important in the process of discontinuing antidepressants.^20,40,45,46^ Furthermore, good therapeutic alliance—a construct measuring aspects of the relation of patient and healthcare provider and closely related to empathy^47^—predicts higher subjective success of antidepressant discontinuation.^26^ The findings of our study are consistent with these findings, demonstrating the association of higher empathy scores with subjective discontinuation success and fewer negative effects.^44^ It is questionable if these arguments fully apply to the withdrawal symptoms experienced after discontinuation of antidepressants. Indeed, the nature of some withdrawal symptoms (brain zaps, akathisia, muscle twitching) seems very specific and unusual as manifestations of psychophysiological processes. An extra argument could be that healthcare professionals do not inform patients very detail about withdrawal effects and therefore do not induce nocebo effects. On the other hand, a relevant nocebo effect might be enhanced by social media messages.

## Conclusion

This study highlights a small, significant positive association between perceived empathy and a successful experience of antidepressant discontinuation by the patient, as evidenced by more subjective success and fewer negative effects of discontinuation. Subjective feeling of success encompasses a broader range of experiences than the binary measure of objective success. For example, dose reduction can relieve side effects or decrease feelings of dependency. Also, feeling acknowledged and supported during the discontinuation process, whether successful or not, can empower patients.^48^ This is relevant because a successful experience regarding antidepressant discontinuation may have a positive influence on the initiation of future attempts.^49^ The small but significant positive association between CARE scores and subjective discontinuation success indicates the potentially valuable role of empathy in the discontinuation process.

Although there are indications of a less important role of psychophysiological processes for the experience of some withdrawal symptoms, we recommend an empathic approach for healthcare providers to support patients during the discontinuation process, next to adequate pharmacological interventions. Integrating patients’ experiences and their intended goals for discontinuation into treatment planning may lead to more personalized and effective discontinuation strategies. To gain a better understanding of the role of non-specific factors in the discontinuation of antidepressants, future studies should measure, in addition to perceived empathy, prior experiences with discontinuation attempts, patients’ expectations and the quality of the shared decision-making process and communication.

## Supplemental Material

sj-docx-2-tpp-10.1177_20451253251397593 – Supplemental material for The role of empathy in antidepressant withdrawal Supplemental material, sj-docx-2-tpp-10.1177_20451253251397593 for The role of empathy in antidepressant withdrawal by Jim van Schie, Peter Lucassen, Tom Birkenhäger, Sjoerd van Belkum, Mariska Bot and Suzanne Ligthart in Therapeutic Advances in Psychopharmacology

sj-pdf-1-tpp-10.1177_20451253251397593 – Supplemental material for The role of empathy in antidepressant withdrawal Supplemental material, sj-pdf-1-tpp-10.1177_20451253251397593 for The role of empathy in antidepressant withdrawal by Jim van Schie, Peter Lucassen, Tom Birkenhäger, Sjoerd van Belkum, Mariska Bot and Suzanne Ligthart in Therapeutic Advances in Psychopharmacology

## Declarations

**Ethics approval and consent to participate:** The Medical Ethical Committee of Amsterdam UMC, Netherlands, determined that the study protocol of OPERA-monitor was exempted from ethical approval according to the Dutch Medical Research Involving Human Subjects Act (WMO) (2019.154). All participants signed digital informed consent forms.

**Consent for publication:** Not applicable.

**Availability of data and materials:** Data and other documents of OPERA-monitor can be provided upon approval of an analysis plan (see for more information opera-project.nl).

## References

[1] Verhaak PFM de Beurs D Spreeuwenberg P. What proportion of initially prescribed antidepressants is still being prescribed chronically after 5 years in general practice? a longitudinal cohort analysis. BMJ Open 2019; 9: e024051. 10.1136/bmjopen-2018-024051 PMC6377556 30813115

[2] Noordam R Aarts N Verhamme KM , et al. Prescription and indication trends of antidepressant drugs in the Netherlands between 1996 and 2012: a dynamic population-based study. Eur J Clin Pharmacology 2015; 71: 369–375. 10.1007/s00228-014-1803-x 25560052

[3] Alabaku O Yang A Tharmarajah S , et al. Global trends in antidepressant, atypical antipsychotic, and benzodiazepine use: a cross-sectional analysis of 64 countries. PLoS ONE 2023; 18: e0284389. 10.1371/journal.pone.0284389 PMC10132527 37099524

[4] Huijbregts KM Hoogendoorn AW Slottje P , et al. Long-term and short-term antidepressant use in general practice: data from a large cohort in the Netherlands. Psychother Psychosom 2017; 86: 362–369. 29131110 10.1159/000480456

[5] Helft starters antidepressiva stopt binnen een halfjaar, https://www.sfk.nl/publicaties/PW/2018/helft-starters-antidepressiva-stopt-binnen-een-halfjaar (accessed 10 June 2025).

[6] GIPdatabank. Vergoeding 2019-2023 voor ATC subgroep N06A: antidepressiva, https://www.gipdatabank.nl/databank?infotype=g&label=00-totaal&tabel_d_00-totaal=B_01-basis&tabel_g_00-totaal=B_01-basis&tabel_h_00-totaal=B_01-basis&geg=vg&spec=&item=N06A

[7] Ambresin G Palmer V Densley K , et al. What factors influence long-term antidepressant use in primary care? findings from the Australian diamond cohort study. J Aff Dis 2015; 176: 125–132. 10.1016/j.jad.2015.01.055 25704565

[8] Cruickshank G MacGillivray S Bruce D , et al. Cross-sectional survey of patients in receipt of long-term repeat prescriptions for antidepressant drugs in primary care. Ment Health Fam Med 2008; 5: 105–109. 22477855 PMC2777559

[9] Piek E Kollen BJ van der Meer K , et al. Maintenance use of antidepressants in Dutch general practice: non-guideline concordant. PLoS One 2014; 9: e97463. 10.1371/journal.pone.0097463 PMC4032267 24858011

[10] Van Leeuwen E van Driel ML Horowitz MA , et al. Approaches for discontinuation versus continuation of long-term antidepressant use for depressive and anxiety disorders in adults. Cochrane Database Syst Rev 2021; 4: CD013495. 10.1002/14651858.CD013495.pub2 PMC8092632 33886130

[11] Framer A. What I have learnt from helping thousands of people taper off antidepressants and other psychotropic medications. Ther Adv Psychopharmacol 2021; 11: 2045125321991274. 10.1177/2045125321991274 PMC7970174 33796265

[12] Van Geffen ECG Brugman M van Hulten R , et al. Patients’ concerns about and problems experienced with discontinuation of antidepressants. Int J Pharm Pract 2007; 15: 291–293.

[13] Henssler J Schmidt Y Schmidt U , et al. Incidence of antidepressant discontinuation symptoms: a systematic review and meta-analysis. Lancet Psychiatry 2024; 13: 526–535. 10.1016/S2215-0366(24)00133-0 38851198

[14] Moncrieff J Hobday H Sørensen A , et al. Evidence on antidepressant withdrawal: an appraisal and reanalysis of a recent systematic review. Psychol Med 2025; 55: e191. 10.1017/S0033291725100652 PMC12315658 40692314

[15] Horowitz MA Framer A Hengartner MP , et al. Estimating risk of antidepressant withdrawal from a review of published data. CNS Drugs 2023; 37: 143–157. 36513909 10.1007/s40263-022-00960-y PMC9911477

[16] Horowitz MA Taylor D. Tapering of SSRI treatment to mitigate withdrawal symptoms. Lancet Psychiatry 2019; 6: 538–546. 30850328 10.1016/S2215-0366(19)30032-X

[17] Berwian IM Walter H Seifritz E , et al. Predicting relapse after antidepressant withdrawal-a systematic review. Psychol Med 2016; 47: 426–437. 27786144 10.1017/S0033291716002580 PMC5244448

[18] Eveleigh R Speckens A van Weel C , et al. Patients’ attitudes to discontinuing not-indicated long-term antidepressant use: barriers and facilitators. Ther Adv Psychopharmacol 2019; 9: 20455125319872344. 10.1177/2045125319872344 PMC6724488 31516691

[19] Maund E Dewar-Haggart R Williams S , et al. Barriers and facilitators to discontinuing antidepressant use: a systematic review and thematic synthesis. J Aff Dis 2019; 245: 38–62. 10.1016/j.jad.2018.10.107 30366236

[20] Bosman RC Huijbregts KM Verhaak PFM , et al. Long-term antidepressant use: a qualitative study on perspectives of patients and GPs in primary care. Br J Gen Pract 2016; 66: e708–e719. 10.3399/bjgp16X686641 PMC5033307 27528709

[21] Crowe M Inder M McCall C. Experience of antidepressant use and discontinuation: a qualitative synthesis of the evidence. J Psychiatr Ment Health Nurs 2023; 30: 21–34. 35713005 10.1111/jpm.12850 PMC10083940

[22] Read J Moncrieff J Horowitz MA. Designing withdrawal support services for antidepressant users: patients’ views on existing services and what they really need. J Psychiatr Res 2023; 161: 298–306. 36989904 10.1016/j.jpsychires.2023.03.013

[23] Read J Lewis S Horowitz MA , et al. The need for antidepressant withdrawal support services: recommendations from 708 patients. Psychiatr Res 2023; 326: 115303. 10.1016/j.psychres.2023.115303 37364505

[24] Wampold BE Imel ZE. The great psychotherapy debate. The evidence for what makes psychotherapy work. New York: Routledge, 2015.

[25] Petrie K Rief W. Psychobiological mechanisms of placebo and nocebo effects: pathways to improve treatments and reduce side effects. Ann Rev Psychol 2019; 70: 599–625. 30110575 10.1146/annurev-psych-010418-102907

[26] White E Read J Julo S. The role of Facebook groups in the management and raising of awareness of antidepressant withdrawal: is social media filling the void left by health services? Ther Adv Psychopharmacol 2021; 11: 2045125320981174. 10.1177/2045125320981174 PMC7816538 33520155

[27] Lincoln TM Sommer D Quazzola M , et al. Predictors of successful discontinuation of antipsychotics and antidepressants. Psychol Med 2023; 53: 3085–3095. 34937582 10.1017/S0033291721005146 PMC10235642

[28] Nienhuis JB Owen J Valentine JC , et al. Therapeutic alliance, empathy, and genuineness in individual adult psychotherapy: a meta-analytic review. Psychother Res 2018; 28: 593–605. 27389666 10.1080/10503307.2016.1204023

[29] Keshtkar L Madigan CD Ward A , et al. The effect of practitioner empathy on patiënt satisfaction. Ann Int Med 2024; 177: 196–209. 38285985 10.7326/M23-2168

[30] Nembhard IM David G Ezzeddine I , et al. A systematic review of research on empathy bin health care. Health Serv Res 2023; 58: 250–263. 35765156 10.1111/1475-6773.14016 PMC10012244

[31] Zhang X Li L Zhang Q , et al. Physician empathy in doctor-patient communication: a systematic review. Health Commun 2024; 39: 1027–1037. 37062918 10.1080/10410236.2023.2201735

[32] Von Elm E Altman DG Egger M , et al. The strengthening the reporting of observational studies in epidemiology (STROBE) statement: guidelines for reporting observational studies. Ann Intern Med 2007; 147: 573–577. 17938396 10.7326/0003-4819-147-8-200710160-00010

[33] Mercer SW Maxwell M Heaney D , et al. The consultation and relational empathy (CARE) measure: development and preliminary validation and reliability of an empathy-based consultation process measure. Family Practice 2004; 21: 699–705. 15528286 10.1093/fampra/cmh621

[34] Van Dijk I Lenferink NSM Lucassen PLBJ , et al. Reliability and validity of the Dutch version of the consultation and relational empathy measure in primary care. Family Practice 2017; 34: 119–124. 27920117 10.1093/fampra/cmw116

[35] Lincoln TM Sommer D Könemund M , et al. A rating scale to inform successful discontinuation of antipsychotics and antidepressants. Psychiatry Res 2012; 298: 113768. 10.1016/j.psychres.2021.113768 33601071

[36] Schober P Boer C Schwarte LA. Correlation coefficients: appropriate use and interpretation. Anesth Analg 2018; 126: 1763–1768. 29481436 10.1213/ANE.0000000000002864

[37] Sauer BC Brookhart MA Roy J , et al. A review of covariate selection for non-experimental comparative effectiveness research. Pharmacoepidemiol Drug Saf 2013; 22: 1139–1145. 24006330 10.1002/pds.3506 PMC4190055

[38] Lewis G Marston L Duffy L , et al. Maintenance or discontinuation of antidepressants in primary care. N Engl J Med 2021; 385: 1257–1267. 34587384 10.1056/NEJMoa2106356

[39] Kishi T Sakuma K Hatano M , et al. Early relapse after antidepressant discontinuation may represent antidepressant discontinuation syndrome in major depressive disorder: a meta-analysis. Asian J Psychiatr 2023; 82: 103502. 36753961 10.1016/j.ajp.2023.103502

[40] Meißner C Meyrose AK Nestoriuc Y. What helps, what hinders antidepressant discontinuation? qualitative analysis of patients’ experiences and expectations. Br J Gen Pract 2024; 74: e466–e474. 10.3399/BJGP.2023.0020 PMC11157563 38228356

[41] Horowitz MA Buckman JEJ Saunders R , et al. Antidepressants withdrawal effects and duration of use: a survey of patients enrolled in primary care psychotherapy services. Psychiatry Res 2025; 350: 116497. 40404538 10.1016/j.psychres.2025.116497

[42] Ruhe H Horikx A van Avendonk MJP , et al. Comment on tapering of SSRI treatment to mitigate withdrawal symptoms. Lancet Psychiatry 2019; 6: 561–562. 31230677 10.1016/S2215-0366(19)30182-8

[43] Groot PC van Os J , Successful use of tapering strips for hyperbolic reduction of antidepressant dose: a cohort study. Ther Adv Psychopharmacol 2021; 11: 20451253211039327. 10.1177/20451253211039327 PMC8404667 34471516

[44] Faasse K Petrie KJ. The nocebo effect: patient expectations and medication side effects. Postgrad Med J 2013; 89: 540–546. 23842213 10.1136/postgradmedj-2012-131730

[45] Nienhuis JB Owen J Valentine JC , et al. Therapeutic alliance, empathy, and genuineness in individual adult psychotherapy: a meta analytic review. Psychother Res 2018; 28: 593–605. 27389666 10.1080/10503307.2016.1204023

[46] Nederlof M Cath DC Stoker LJ , et al. Guidance by physicians and pharmacists during antidepressant therapy: patients’ needs and suggestions for improvement. BMC Psychiatry 2017; 7: 388. 10.1186/s12888-017-1522-9 PMC5716388 29202819

[47] Van Leeuwen E Anthierens S van Driel ML , et al. Should I, can I, dare I? Patients’ view on stopping long-term antidepressant use, a qualitative study. Acta Clin Belg 2022; 77: 962–969. 35007191 10.1080/17843286.2021.2024384

[48] White E. Tapering antidepressants: why do tens of thousands turn to Facebook groups for support? Br J Gen Pract 2021; 71: 315. 34319884 10.3399/bjgp21X716309 PMC8249018

[49] Rutherford BR Wager TD Roose SP. Expectancy and the treatment of depression: a review of experimental methodology and effects of patient outcome. Curr Psychiatry Rev 2014; 6: 1–10. 10.2174/157340010790596571 PMC4011659 24812548

