# Supplementary material for: The role of empathy in antidepressant withdrawal
Source: Ther Adv Psychopharmacol. 2025 Nov 25;15:20451253251397593. doi: 10.1177/20451253251397593 (PMC12647550; doi:10.1177/20451253251397593)
Supplement: sj-docx-2-tpp-10.1177_20451253251397593 – Supplemental material for The role of empathy in antidepressant withdrawal [file sj-docx-2-tpp-10.1177_20451253251397593.docx]

Supplemental table 1. Comparison of participants not included in analyses vs. participants included in the analyses

|  | Not included (n=765) | Included (n=153) | p |
| --- | --- | --- | --- |
| Age in years, mean (SD) | 40.7 (14.6) | 44.4 (14.8) | 0.005 |
| Sex, N (%)* |  |  | 0.490 |
| Male | 256 (33.6%) | 47 (30.7%) |  |
| Female | 506 (66.4%) | 106 (69.3%) |  |
| Education level |  |  | 0.377 |
| Low | 124 (16.2%) | 18 (11.8%) |  |
| Medium | 285 (37.3%) | 61 (39.9%) |  |
| High | 356 (46.5%) | 74 (48.4%) |  |
| Partner status |  |  | 0.598 |
| No partner | 267 (34.9%) | 50 (32.7%) |  |
| With partner | 498 (65.1%) | 103 (67.3%) |  |
| Type of antidepressant |  |  | 0.124 |
| Sertraline | 311 (40.7%) | 52 (34.0%) |  |
| Citalopram | 454 (59.3%) | 101 (66.0%) |  |
| Dose |  |  | 0.539 |
| Below MED | 137 (17.9%) | 33 (21.6%) |  |
| At MED | 409 (53.5%) | 80 (52.3%) |  |
| Above MED | 219 (28.6%) | 40 (26.1%) |  |
| Number of months with depression in lifetime |  |  | 0.874 |
| <12 | 217 (28.4%) | 41 (26.8%) |  |
| 12 to 36 | 342 (44.7%) | 68 (44.4%) |  |
| >36 | 206 (26.9%) | 44 (28.8%) |  |
| Psychiatric comorbidity |  |  | 0.324 |
| 0 | 282 (36.9%) | 59 (38.6%) |  |
| 1 to 2 | 359 (46.9%) | 63 (41.2%) |  |
| 3 or more | 124 (16.2%) | 31 (20.3%) |  |
| Comedications |  |  | 0.268 |
| 0 | 425 (55.6%) | 85 (55.6%) |  |
| 1 to 2 | 239 (31.2%) | 41 (26.8%) |  |
| 3 or more | 101 (13.2%) | 27 (17.6%) |  |

*3 participants in the Not included group had sex = other; these were excluded from this analysis.

Supplemental table 2. Overview of linear and logistic regression models with the CARE Score as independent variable and DSS Success, DSS Positive Effects, DSS Negative Effects, and Objective Success as outcome variables in the subsample who had a discontinuation attempt in the past 2 years (n=107)

|  | DSS Success | | | DSS Positive Effects | | | DSS Negative Effects | | | Objective Success | | |
| --- | --- | --- | --- | --- | --- | --- | --- | --- | --- | --- | --- | --- |
|  | **B** | **CI (95%)** | **p** | **B** | **CI (95%)** | **p** | **B** | **CI (95%)** | **p** | **OR** | **CI (95%)** | **p** |
| Model (covariates) |  |  |  |  |  |  |  |  |  |  |  |  |
| CARE score | **.182** | **.036 to .327** | **.015** | **.250** | **.079 to .420** | **.004** | **-.253** | **-.443 to -.063** | **.010** | 1.007 | .963 to 1.052 | .766 |
| CARE score (+ demographic) | **.166** | **.020 to .318** | **.026** | **.249** | **.076 to .422** | **.005** | **-.234** | **-.424 to -.043** | **.017** | 1.004 | .959 to 1.051 | .747 |
| CARE score (+ demographic + clinical) | **.129** | **.012 to .321** | **.035** | **.275** | **.096 to .454** | **.003** | **-.239** | **-.433 to -.046** | **.016** | 1.002 | .954 to 1.054 | .926 |

*Note:* **demographic covariates** = sex, age, marital status, and education level; **clinical covariates** = type of AD, dosage compared to MED, cumulative duration of depression, number of psychiatric comorbidities, and number of comedications; **B =** Regression Coefficient; **CI** = Confidence Interval; **OR** = Odds Ratio; **p** = p-value. Significant effects are printed in bold
